# Strategy and model building in the fourth dimension: a null model for genotype × age interaction as a Gaussian stationary stochastic process

**DOI:** 10.1186/1471-2156-4-S1-S34

**Published:** 2003-12-31

**Authors:** Vincent P Diego, Laura Almasy, Thomas D Dyer, Júlia MP Soler, John Blangero

**Affiliations:** 1 Department of Genetics, Southwest Foundation for Biomedical Research, San Antonio, Texas, USA; 2 Department of Anthropology, Binghamton University, State University of New York, Binghamton, New York, USA; 3 Department of Statistics, University of Sao Paulo, Sao Paulo SP, Brazil

## Abstract

**Background:**

Using univariate and multivariate variance components linkage analysis methods, we studied possible genotype × age interaction in cardiovascular phenotypes related to the aging process from the Framingham Heart Study.

**Results:**

We found evidence for genotype × age interaction for fasting glucose and systolic blood pressure.

**Conclusions:**

There is polygenic genotype × age interaction for fasting glucose and systolic blood pressure and quantitative trait locus × age interaction for a linkage signal for systolic blood pressure phenotypes located on chromosome 17 at 67 cM.

## Background

The Framingham Heart Study (FHS) [1] offers a unique opportunity to assess possible genotype × age (G × age) interaction in the genetic architecture of complex traits. To study G × age interaction in the FHS, we built on the variance components model [2], which, assuming negligible dominance, may be given as:



where Σ is the variance-covariance matrix of a pedigree,  is a matrix of estimated elements, , giving the proportion of alleles identical by descent (IBD) in individuals i and j at a quantitative trait locus (QTL), Φ is the kinship matrix of the pedigree, **I **is the identity matrix, and , , and  are variances of the additive QTL, polygenic and environmental factors, respectively.

Our study was carried out in two phases. In Phase 1, we made inferences on polygenic G × age interaction using what we call the G × age model [3]. Also, we experiment with correlation functions in the G × age model. In Phase 2, we implement the QTL version of this model. Multivariate variance components linkage analysis [4] can also be used to detect G × age interaction. We show how the bivariate variance components model can be used to detect QTL × age interaction. Given that the G × age and bivariate models apply to cross-sectional and longitudinal data, respectively, we compare their relative abilities to detect G × age interaction.

## Models and Methods

Using the bivariate mixed model [5], Blangero [6] derived the following expression for the genetic variance in phenotype response to two different environments:



where  and  are the additive genetic variances of the trait in Environments 1 and 2, in this case two ages,  is the additive genetic variance of the phenotypic difference between environments (i.e., trait response), and ρ_G _is the genetic correlation between environments, in this case ages. The term environment, denoted by E, is used in accordance with standard statistical genetic theory [6]. There is no G × E interaction (i.e.,  = 0) if  =  =  and ρ_G _= 1 [6]. To model  = 0,  and ρ_G _are parameterized as functions of age, which is the environment of interest in our analyses:



where  is parameterized as an exponential function [7] and ρ_G _as exponential decay [8], and α, β, and λ are parameters to be estimated, and m and n are two ages. Thus, the null hypotheses are β = 0 and λ = 0, which reflect a stationary covariance function.

In our analyses, we used the expected covariance matrix for a given pedigree [3], which has elements giving the covariance in trait value *y *for any two relatives:



where x and z index individuals at ages m and n, respectively, Φ_*xz *_gives their kinship status, π is the probability that a random allele is IBD at the QTL, δ_xz _is 1 for x = z and 0 otherwise, subscripts g, q, and e denote the polygenic, QTL, and environmental components, respectively, and average age is taken over the sample population, defined as all individuals measured for the trait of interest. Some points of clarification are needed. This is a cross-sectional model that applies generally to three types of pair-wise comparisons of individuals. In one type, let x = z such that *m *= *n*. Equation (5) gives the variances in this situation, in accord with the standard variance components model. In a second type, it may be such that x ≠ z while m ≠ n, and, in a third type, it may be such that x ≠ z while m ≠ n. Note that none of these types are longitudinal comparisons, which would be the case where x = z while m ≠ n (i.e., the same individual is measured at different ages). The goal of this approach lies in estimation of the change parameters, namely β and λ, so that we can test the null hypotheses stated above.

For the bivariate model, a trait measured at two different time points is treated as a bivariate phenotype. The pedigree covariance matrix model for a bivariate phenotype K, with constituent phenotypes I and J, may be written as [4]:

**Σ_K _= 2Φ****G + ****Q + I****E, **    (6)

where the new matrices **G**, **Q**, and **E **convey the polygenic, QTL, and environmental variance components, respectively, and  is the Kronecker-product operator. The variance components for this model are those for the univariate model for traits I and J, given by Σ_I _and Σ_J _(cf. equation (1)), and the trait cross-covariances, which may be parameterized as ρ_gij_σ_gi_σ_gj_, ρ_qij_σ_qi_σ_qj_, and ρ_eij_σ_ei_σ_ej _for the polygenic, QTL, and environmental components, respectively [4]. The null hypotheses of no polygenic genotype or QTL × age interaction are expressed as ρ_gij _= 1 and ρ_qij _= 1, respectively.

Scatter plots were examined for traits showing increasing or decreasing variance in trait values with age, suggesting potential for G × age interaction. Traits meeting these criteria, namely systolic blood pressure (SBP) and fasting glucose (GLUC), were analyzed using SOLAR [2], with age, sex, hypertension medication, and body mass index (BMI) as covariates. Phase 1 analyses were conducted on an augmented data set. Exams 12 (1970), 16 (1978), 18 (1982), and 20 (1986) in Cohort 1 were combined with Exams 1 (1971), 2 (1979), 3 (1983), and 4 (1987) in Cohort 2, respectively. To reduce kurtosis after combining, a few outliers were removed for SBP (1–6 per exam, all with values > the mean) but many for GLUC (51–101 per exam, all but one > the mean). No data transformation was made. Since diabetic status was not available, we could not control for it. This gave combined measurement periods 1–4.

Based on genome scans that we conducted for both SBP and GLUC, we decided to focus on SBP. For Phase 2, data for SBP values corrected for hypertension treatment for Cohorts 1 and 2, imputed following Levy et al. [9] (see Soler and Blangero [10]), were analyzed. Bivariate analyses were performed on residuals from multiple regressions with corrected SBP values as the dependent variable and age, sex and BMI as independent variables. For the bivariate analyses, analysis of residuals was necessary due to the unbalanced and incomplete structure of the FHS longitudinal data, which made covariate specification unrepresentative across measurements. For this SBP data set (corrected values and residuals), we experimented with combined exams from Cohorts 1 and 2 and with exams from Cohort 2 taken separately.

## Results

### Phase 1

**Figure 1:**
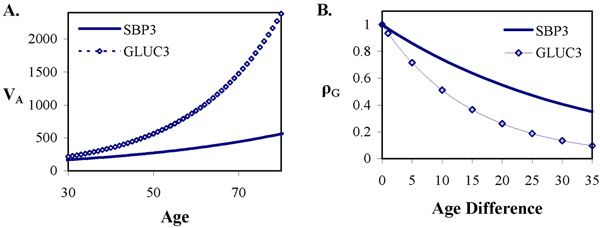
**Genetic parameters, combined measurement period 3. **A, Variance functions for SBP (α = 4.412 ± 0.213, *p *< 0.001; β = 0.024 ± 0.009, *p *= 0.019) and GLUC (α = 3.937 ± 0.202, *p *< 0.001; β = 0.048 ± 0.010, *p *= 0.003). B, Correlationfunctions for SBP (λ = 0.030 ± 0.016, *p *= 0.016) and GLUC (λ = 0.067 ± 0.024, *p *< 0.001).

We implemented equation (5) using three different correlation functions, specifically the standard normal, Cauchy, and exponential. These gave consistent results (not shown). Only selected results for the exponential function given by equation (4) are reported. For SBP, only combined measurement period 3 gave strong evidence for polygenic G × age interaction (Fig. 1). For GLUC, combined measurement periods 2–4 all gave strong evidence for polygenic G × age interaction and we believe combined measurement period 3 to be representative (Fig. 1).

The *p*-values reported in Figure 1 and Tables 1,2 are for the likelihood ratio statistic: Λ = -2 [ln L(θ_N_)- ln L(θ_A_)], where the null, H_N _(parameter constrained to 0 or 1 as appropriate), is compared to the alternative, H_A _(parameter estimated). In general, Λ is distributed as a χ^2 ^random variable with degrees of freedom (d.f.) equal to the difference in the number of parameters under the null and alternative hypotheses [11]. If parameter values are constrained to a boundary under the null hypothesis, the asymptotic distribution of Λ is given by a mixture of  random variables, where n denotes the d.f. and where the mixture may include n = 0 (point mass at 0) [11]. However, the traditional criterion (i.e., difference in parameters) is conservative [3].

### Phase 2

**Figure 2:**
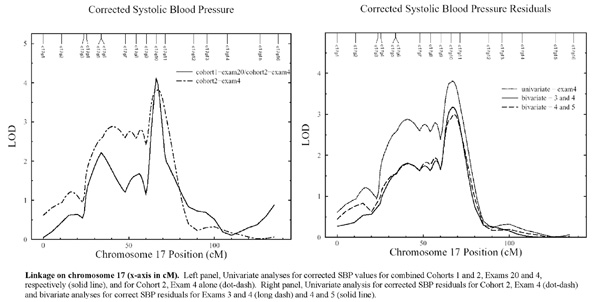
**Linkage on chromosome 17 (x-axis in cM). **Left panel, Univariate analyses for corrected SBP values for combined Cohorts 1 and 2, Exams 20 and 4, respectively (solid line), and for Cohort 2, Exam 4 alone (dot-dash). Right panel, Univariate analysis for corrected SBP residuals for Cohort 2, Exam 4 (dot-dash) and bivariate analyses for corrected SBP residuals for Exams 3 and 4 (long dash) and 4 and 5 (solid line).

We recovered the linkage signal for SBP on chromosome 17 at 67 cM reported by the FHS group [9] (Fig. 2; all maximum LOD scores > 3). We then applied the G × age and bivariate models in search of QTL × age interaction. Table 1 contains model selection criteria [12] and selected likelihood ratio tests for univariate analyses of corrected SBP values using the G × age model for combined Cohorts 1 and 2, Exams 20 and 4, respectively. Table 2 contains similar information for bivariate analyses of corrected SBP residuals using the bivariate model for Cohort 2, Exams 4 and 5.

## Conclusion

Our results suggest that there is polygenic G × age interaction for both GLUC and SBP (Fig. 1; Tables 1,2) and QTL × age interaction for the putative QTL for SBP phenotypes on chromosome 17 at 67 cM (linkage: Fig. 2; interaction: Table 1). To our knowledge, this is the first demonstration of QTL × age interaction in humans using linkage analysis methods.

The cross-sectional and longitudinal analyses do not give the same results. The difference may mean that cross-sectional data are better than longitudinal data at capturing G × age interaction. However, based on the received wisdom regarding the relative utility of cross-sectional and longitudinal analyses [13], this explanation does not seem tenable. Alternatively, that the bivariate model did not detect QTL × age interaction (Table 2) may simply be due to the loss in power with an overly parameterized model [12]. Another explanation is that the bivariate model is really operating on time rather than on age and so perhaps the brief time span between Cohort 2, Exams 4 and 5, was too short to capture an interaction effect. Yet another explanation of the difference between the cross-sectional and longitudinal analyses is that the latter was carried out on Cohort 2 only, which, taken by itself, yielded a smaller sample size. Lastly, there is the possibility of some mixture of the last three problems.

Given that equations (5) and (6) derive from the same underlying modeling framework – namely variance components – they can be conceptualized as a strategy for testing the null hypothesis that a given phenotype "translated along the time axis" is a Gaussian covariance stationary stochastic process [14]. This is now an established approach in statistical genetics [14], and is readily extended to the genetics of aging. It is our hope that the analyses herein contribute to a better understanding of the genetic architecture of the complex traits associated with the aging process and associated complex diseases (e.g., cardiovascular disease). We conclude that the G × age and bivariate models offer a feasible system for model building in the fourth dimension.

## Figures and Tables

**Table 1:** Univariate Analyses for Combined Cohort 1, Exam 20 and Cohort 2, Exam 4

Models^A^	Ln Likelihood	AIC^B^	Evidence Ratio^C^	*p*-Value	Ratio Test^D^
1. Polygenic (2)	-6714.557	13433.11	5.7 × 10^77^	1.35 × 10^-5^	1 vs. 6
2. P × age (5)	-6537.371	13084.74	128.8433	1.69 × 10^-76^	1 vs. 2
3. Conα G (4)	-6583.193	13174.39	3.77 × 10^21^	1.04 × 10^-21^	3 vs. 2
4. Conβ G (4)	-6542.711	13093.42	9888.592	0.00108	4 vs. 2
5. Conλ (4)	-6538.801	13085.6	198.1198	0.09078	5 vs. 2
6. QTL (3)	-6705.086	13416.17	1.2 × 10^74^	2.68 × 10^-74^	6 vs. 7
7. QTL × age (7)	-6530.512	13075.02	1	0.00105	2 vs. 7
8. Conα QTL (6)	-6534.916	13081.83	30.09494	0.00300	8 vs. 7
9. Conβ QTL (6)	-6533.796	13079.59	9.819076	0.01038	9 vs. 7

^A^Models: QTL: model for polygenic + QTL component. P × age model: polygenic G × age. Con: Constrained parameter for P × age, Q × age, or bivariate model, where the parameters may be α, β, λ, or ρ and where the suffix G indicates the polygenic component. The numbers of parameters per model are in parentheses following each one. ^B^AIC: Akaike's Information Criterion = -2 Ln L (θ| data) + 2K, where θ is a parameter vector and K is the number of parameters. ^C^Evidence Ratio: w_min_/w_i _= exp(Δ_i_/2), where w_min _is set to 1, w_i _= [exp(-Δ_i _/2)]/Σ_r = 1_exp(-Δ_r_/2) and Δ_i _= AIC_i _- AIC_min_. ^D^Models compared.

**Table 2:** Bivariate Analyses – Cohort 2, Exams 4 and 5 Residuals^A^

Models	Ln Likelihood	AIC	Evidence Ratio	*p*-Value; Ratio Test	Ratio Test
1. Biv-polyg (6)	-8940.4	17888.81	15248.48	0.00024	1 vs. 2
2. Biv-QTL (9)	-8930.77	17869.54	1	-----	-----
3. Conρ G (8)	-8940.4	17888.81	15248.48	1.14 × 10^-5^	3 vs. 2
4. Conρ Q (8)	-8931.25	17870.49	1.608439	0.32815	4 vs. 2

^A^See footnotes to Table 1.
